# Multiscale attention-over-attention network for retinal disease recognition in OCT radiology images

**DOI:** 10.3389/fmed.2024.1499393

**Published:** 2024-11-08

**Authors:** Abdulmajeed M. Alenezi, Daniyah A. Aloqalaa, Sushil Kumar Singh, Raqinah Alrabiah, Shabana Habib, Muhammad Islam, Yousef Ibrahim Daradkeh

**Affiliations:** ^1^Department of Electrical Engineering, Faculty of Engineering, Islamic University of Madinah, Madinah, Saudi Arabia; ^2^Department of Information Technology, College of Computer, Qassim University, Buraydah, Saudi Arabia; ^3^Department of Computer Engineering, Marwadi University, Rajkot, Gujarat, India; ^4^Department of Electrical Engineering, College of Engineering, Qassim University, Buraydah, Saudi Arabia; ^5^Department of Computer Engineering and Information, College of Engineering in Wadi Alddawasir, Prince Sattam Bin Abdulaziz University, Al-Kharj, Saudi Arabia

**Keywords:** retinal recognition, OCT imaging, attention mechanism, deep learning, medical imaging, multi-level features

## Abstract

Retinal disease recognition using Optical Coherence Tomography (OCT) images plays a pivotal role in the early diagnosis and treatment of conditions. However, the previous attempts relied on extracting single-scale features often refined by stacked layered attentions. This paper presents a novel deep learning-based Multiscale Feature Enhancement via a Dual Attention Network specifically designed for retinal disease recognition in OCT images. Our approach leverages the EfficientNetB7 backbone to extract multiscale features from OCT images, ensuring a comprehensive representation of global and local retinal structures. To further refine feature extraction, we propose a Pyramidal Attention mechanism that integrates Multi-Head Self-Attention (MHSA) with Dense Atrous Spatial Pyramid Pooling (DASPP), effectively capturing long-range dependencies and contextual information at multiple scales. Additionally, Efficient Channel Attention (ECA) and Spatial Refinement modules are introduced to enhance channel-wise and spatial feature representations, enabling precise localization of retinal abnormalities. A comprehensive ablation study confirms the progressive impact of integrated blocks and attention mechanisms that enhance overall performance. Our findings underscore the potential of advanced attention mechanisms and multiscale processing, highlighting the effectiveness of the network. Extensive experiments on two benchmark datasets demonstrate the superiority of the proposed network over existing state-of-the-art methods.

## 1 Introduction

The healthcare landscape has undergone a remarkable transformation in recent years, driven by groundbreaking technological advancements that complement and amplify human medical expertise. This evolution is particularly evident in diagnostics and patient care, where computational methods have become increasingly pivotal. Among these innovations, machine learning (ML) techniques, especially those under deep learning (DL), have demonstrated an exceptional ability to analyze complex medical data with a level of precision and speed that was previously unattainable. These sophisticated algorithms excel at identifying intricate patterns within vast datasets, offering new insights that can significantly enhance diagnostic accuracy and treatment efficacy. The impact of these technological strides is particularly pronounced in medical imaging, where the interpretation of nuanced visual information is paramount for early and accurate diagnosis. In the domain of ophthalmology, where the preservation of vision is of utmost importance, these advancements have ushered in a new era of diagnostic capabilities, promising to revolutionize how eye diseases are detected, monitored, and treated.

The human eye is a complex and sensitive organ that allows us to see and interact with our surroundings in various ways. The significance of vision extends far beyond mere sight; it is inextricably linked to the quality of human life. When visual impairment occurs, its repercussions can be far-reaching, affecting not only an individual's ability to navigate their surroundings but also their sense of autonomy and capacity to engage in daily activities. According to the World Health Organization, at least 2.2 billion people have a near or distant vision impairment, with at least 1 billion of these cases being preventable or yet to be addressed (1). These statistics emphasize the pressing requirement for greater accessibility to eye care services and therapies leading to assisted living (2). The growing need for prompt action in eye care and the inclusion of advanced computer technologies for evaluating OCT visuals indicates an important step forward in diagnosis. OCT is a non-invasive imaging technique that has long been essential to diagnosing and treating various eye disorders. It provides high-resolution, cross-sectional visualizations of retinal structures. Interpreting these complex images sometimes presents difficulties even for experienced practitioners since minor anomalies may be readily missed. The use of advanced algorithms for image processing on OCT data confronts this challenge, thereby providing the ability to identify subtle alterations suggestive of early-stage ocular disorders that may remain undetected.

Spectral-domain OCT (SD-OCT) presents important clues for ocular disorders. Significant developments in computer-assisted imaging in SD-OCT were made, particularly by incorporating AI and DL methodologies. These improvements have enhanced ophthalmologists' capacity to make swift and appropriate diagnoses about the evolution of macular degeneration. Recent research has shown the effectiveness of AI-driven techniques in OCT image analysis. For instance, Diaz et al. (3) developed an entirely automated method for recognizing and segmenting the foveal avascular zone in OCT-A images. The method showed a significant association of 0.93 with expert manual measures when validated on 213 images of healthy and diabetic subjects. It exhibited excellent results with Jaccard indices of 0.82 for healthy and 0.83 for diabetic images. The study Stanojević et al. (4) investigated several CNN architectures for classifying retinal diseases. Their Inception-based algorithm attained optimal performance, with a success rate of 95.528% after hyperparameter adjustment. To tackle noise problems and resolution constraints in OCT image classification, Opoku et al. (5) introduced a CLAHE-CapsNet model. The model attained an overall accuracy of 97.7% and a precision of 99.3% using the UCSD dataset, which comprises 84,495 images distributed across four categories. A dual guidance DL framework for diagnosing Age-related Macular Degeneration was developed, including a CM-CNN for categorization and a CAM-UNET for segmentation. Their model achieved an accuracy of 96.93% and a Dice coefficient of 77.51% for segmentation when evaluated on the UCSD dataset. The study Hassan et al. (6) advanced the field by introducing the EOCT approach to retinal OCT image classification, which integrates a modified ResNet-50 with random forest methods. The model exhibited exceptional performance, with 97.47% accuracy and 98.36% sensitivity, surpassing standard pre-trained models. The study Udayaraju et al. (7) proposed a Hybrid Multilayered Classification (HMLC) system that combines CNN and VGG-19 models for classifying four retinal disorders. The HMLC leveraged advanced features from both models, demonstrating high classification accuracy across various performance metrics. These studies collectively highlight the growing potential of DL techniques in OCT image analysis for retinal disease recognition, showcasing improvements in accuracy, efficiency, and clinical applicability.

### 1.1 Limitations of the existing studies

Despite the significant advancements in OCT image analysis for retinal disease recognition, several limitations persist in existing approaches. Many current models rely on single-scale feature extraction, potentially missing crucial information at different levels of abstraction (8). The lack of effective attention mechanisms tailored specifically for OCT images hinders the ability to focus on the most relevant features across various scales. Additionally, most architectures must adequately address the challenge of capturing highly representative contextual information, which is crucial for accurate disease classification. Integrating advanced attention mechanisms and spatial processing techniques is often limited or absent in many existing models. Furthermore, the complexity of retinal structures and the subtle nature of disease-related changes in OCT images necessitate more advanced feature extraction and optimal decoding approaches. These limitations underscore the need for a more comprehensive and adaptive approach to OCT image analysis that can effectively leverage multiscale features and incorporate advanced attention mechanisms. Additionally, such an approach must balance local and global information processing to improve retinal disease recognition.

### 1.2 Contributions

In this work, we propose a novel DL network for retinal disease recognition using OCT images. Our approach addresses the limitations of existing methods by incorporating advanced feature extraction techniques, attention mechanisms for feasible layers, and multi-scale processing. The key contributions of our work are as follows:

- We obtained multi-level features from an EfficientNetB7 backbone to gather information across many levels of abstraction rather than relying on single-scale features. This strategy enables a more comprehensive representation, allowing the network to identify complex and multi-structure patterns essential for retinal diseases.
- We introduced a novel Pyramidal Attention mechanism that integrates Multi-Head Self-Attention (MHSA) and Dilated Atrous Spatial Pyramid Pooling (DASPP) modules. This approach allows our network to jointly capture long-range relationships and multi-scale contexts to improve its capacity to focus on the most important features across various scales.
- Our network utilizes three mature feature maps using dilated convolutions (DConv) at diverse dilation rates, enabling the network to focus on context across several receptive fields. This multi-tiered architecture allows our network to proficiently assess localized features and overarching structures, which is essential for precise disease identification.
- We included advanced components, including Efficient Channel Attention (ECA) and Spatial Refinement modules, to improve our network's performance. The ECA module enhances the channel-wise representation of features by adaptively analyzing the significance of each channel without requiring dimensionality reduction, hence preserving essential information. Additionally, the Spatial Refinement module enhances spatial details by refining mapping features. This deliberate integration of channel and spatial attention processes allows the network to more efficiently simulate complex feature interactions, leading to enhanced accuracy.

The remaining sections of the study are organized as below: Section 2 presents the review of the research work on a similar topic. Section 3 explained the detailed methodology and each component of the proposed network. Section 4 shows the empirical findings and ablation study. Moreover, the comparative analysis with other methods is explained in more detail. Section 5 technically discusses the aspects of the proposed network and the reasons for superior performances. Finally, Section 6 concludes the paper with future research directions.

## 2 Related literature

The emergence of ML, specifically DL, has completely transformed the many fields of disease diagnosis and monitoring (9, 10), leading to sustainable developments (11). Similarly, visual intelligence has transformed the field of retinal disease recognition using image processing (12). Recently, the research community has focused on OCT image categorization, which can be classified into two primary domains: classical ML and DL. As shown in Table 1, a yearly literature review of related studies on OCT analysis reveals the utilization of different approaches. For instance, the study by Oliveira et al. (13) proposed an extension of work on drusen detection, incorporating new features such as distance between limiting boundaries and wavelet coefficients, along with multi-label classification. Their method improved upon previous results, achieving higher AUC and Dice coefficient scores. It demonstrated the ability to identify individual drusen within clusters, potentially allowing for more detailed AMD staging based on drusen size. Another study by Habib et al. (14) proposed three automated methods for drusen segmentation based on U-Net convolutional neural networks. Their best-performing approach involved training the CNN to segment Bruch's membrane and RPE, followed by a post-processing step combining shortest path finding and polynomial fitting to detect drusen. When validated on a large dataset of over 50,000 annotated images, the method demonstrated superior accuracy compared to existing state-of-the-art methods. The study by Asgari et al. (15) proposed a novel multi-decoder network architecture for automated drusen segmentation in OCT scans. Their approach treated Dusen segmentation as a multitasking problem, using separate decoders for each target class (OBRPE and BM) and an additional decoder for the area between layers, with inter-decoder connections for improved regularization. The method demonstrated superior performance compared to baselines in both layer and drusen segmentation tasks when validated on diverse AMD and control OCT datasets. Wang et al. (16) proposed a novel multi-scale transformer global attention network (MsTGANet) for drusen segmentation in retinal OCT images, incorporating multi-scale transformer non-local modules and multi-semantic global attention, along with a semi-supervised version (Semi-MsTGANet) to leverage unlabeled data, demonstrating superior performance compared to state-of-the-art CNN-based methods.

**Table 1:** Yearly literature review of the related studies under the umbrella of OCT analysis.

**References**	**Dataset**	**Images used**	**Method**	**Accuracy %**	**Year**
Rasti et al. (17)	OCT	108,312	Multi-scale ensemble CNN model	98.8	2017
Lu et al. (18)	OCT (Manually labeled)	25,134	ResNet 101 Layered	95.9	2018
Díaz et al. (3)	OCT	Limited dataset	Image processing techniques for automatic segmentation and extraction	93	2019
Khan et al. (9)	OCTX	84,484	Deep ensemble network	98.53	2020
Aloraini et al. (19)	OCT	Entire dataset	Deep recurrent residual inception network	98.8	2020
Kim and Tran (20)	OCT	108,309	ResNet152	98.8	2020
Rahimzadeh and Mohammadi (21)	OCT and OCTID	3,213 and entire dataset	Deep ensemble CNN	96.4 and 98.70	2021
Subramanian et al. (22)	OCT C8	24,000	VGG16, VGG19, Densenet201, and InceptionV3	97	2022
Stanojević et al. (4)	OCT V2	V2-dataset	Deep CNN based on Inception architecture	95.55	2023
Hassan et al. (6)	OCT	Entire dataset	Random forest models with ResNet 50	96.93	2023
Diao et al. (23)	OCT	Entire dataset	Mask guided CNN	96.93	2023
Opoku et al. (5)	OCT	84,495	Capsule network with Adaptive histogram equalization	97.7	2023
Udayaraju et al. (7)	OCT	5,000 for train/test	Hybridmultilayered classification CNN-VGG19.	97.7	2023
Naik et al. (24)	OCT	108,312	InceptionV3 and Xception with self attention	96.6	2024
Yang et al. (25)	OCT	Subset of dataset	Ensemble model based on CNN, EfficientNet_v2, and ResNet	97.89	2024

More recent DL and attention approaches include (26, 27), which introduced the Informative Attention Convolutional Neural Network (IA-net) for automatic CNV segmentation in OCT images. This network features an attention enhancement block and novel informative loss to improve small CNV detection and overall segmentation accuracy, outperforming traditional methods in experimental evaluations. Zhang et al. (28) proposed a multi-scale parallel branch CNN (MPB-CNN) for CNV segmentation in SD-OCT images, featuring atrous convolution, intra- and inter-branch connections, and gradient-constrained loss, achieving reliable segmentation results with mean dice value of 0.757 and overlap ratio of 60.8% in cross-validation experiments. Meng et al. (29) introduced a multi-scale information fusion network (MF-Net) for CNV segmentation in retinal OCT images, incorporating a multi-scale adaptive-aware deformation module and semantics-details aggregation module, along with a semi-supervised version (SemiMF-Net), demonstrating superior performance compared to state-of-the-art algorithms in comprehensive experiments. Wang et al. (30) proposed a two-stream CNN for multi-modal AMD categorization using fundus and OCT images, introducing Loose Pair training and extending class activation mapping for visual interpretation, demonstrating improved performance over traditional methods in real-world clinical data experiments. Kermany et al. (31) applied a transfer learning algorithm using InceptionV3 pre-trained on ImageNet for diagnosing retinal OCT images, addressing the critical need for automated analysis in conditions like AMD and diabetic macular edema, which are leading causes of blindness and require frequent OCT-guided anti-VEGF therapy. Similarly, the authors in (32, 33) introduced a novel dimensionality reduction algorithm for Cholangiocarcinoma hyperspectral images and convolution transformer for hyperspectral image classification.

The study by Fang et al. (34) introduced a lesion-aware CNN for retinal OCT image classification, incorporating a lesion detection network to generate attention maps, guiding the classification network to focus on lesion-related regions, demonstrating improved efficiency and effectiveness on two clinical OCT datasets. Khan et al. (9) presented a technique for detecting and classifying OCT images into three distinct categories: DME, CNV, and DRUSEN, as well as normal Retina. The OCTx model is an improved version of an ensemble model designed specifically for diagnosing eye illnesses using OCT data. Nevertheless, the existing suggested model underwent training and testing using a particular dataset, which may diminish its ability to classify data in real-world scenarios accurately. Farman et al. (35) proposed a novel multiscale CNN architecture for AMD diagnosis, featuring seven convolutional layers and multiscale convolution, achieving high accuracy across multiple datasets (99.73% on Mendeley, 98.08% on OCTID, 96.66% on Duke, 97.95% on SD-OCT Noor) and demonstrating potential for real-time implementation in rapid eye screening. Sotoudeh-Paima et al. (36) proposed a multi-scale CNN based on a feature pyramid network for AMD diagnosis, incorporating feature fusion across convolutional blocks to capture inter-scale variations, demonstrating superior performance over existing frameworks and improved accuracy through gradual learning on large OCT datasets, with potential for clinical use as a screening tool. Ma et al. (37) introduced a hybrid ConvNet-Transformer network (HCTNet) for retinal OCT image classification, combining residual dense blocks for low-level feature extraction with parallel Transformer and ConvNet branches for global and local context, achieving superior accuracy (91.56% and 86.18% on two public datasets) compared to pure ViT and ConvNet-based methods.

Most recently, Rahil et al. (38) presented a technique for identifying and separating three distinct retinal conditions, namely cysts. The author used a deep ensemble-based design with a modified version of the UNET architecture. Additionally, a predictor block was implemented for the ensemble technique to consolidate the outcomes of all three models. Although their model achieved a classification accuracy of 79%, it may be enhanced and optimized by using an ensemble method. Mehta et al. (23) proposed a dual guidance DL framework for AMD diagnosis, featuring a complementary mask-guided CNN (CM-CNN) for classification and a class activation map guided UNet (CAM-UNet) for segmentation, achieving 96.93% classification accuracy and 77.51% Dice coefficient for segmentation on the UCSD dataset, outperforming existing single-task and multi-task networks. The study by Khalil et al. (8) presented inception backbone guided modified dual attention network. However, existing approaches in the literature predominantly emphasize single-scale features, often lacking the necessary refinement to capture complex, multi-scale information effectively.

## 3 Methodology

**Figure 1:**
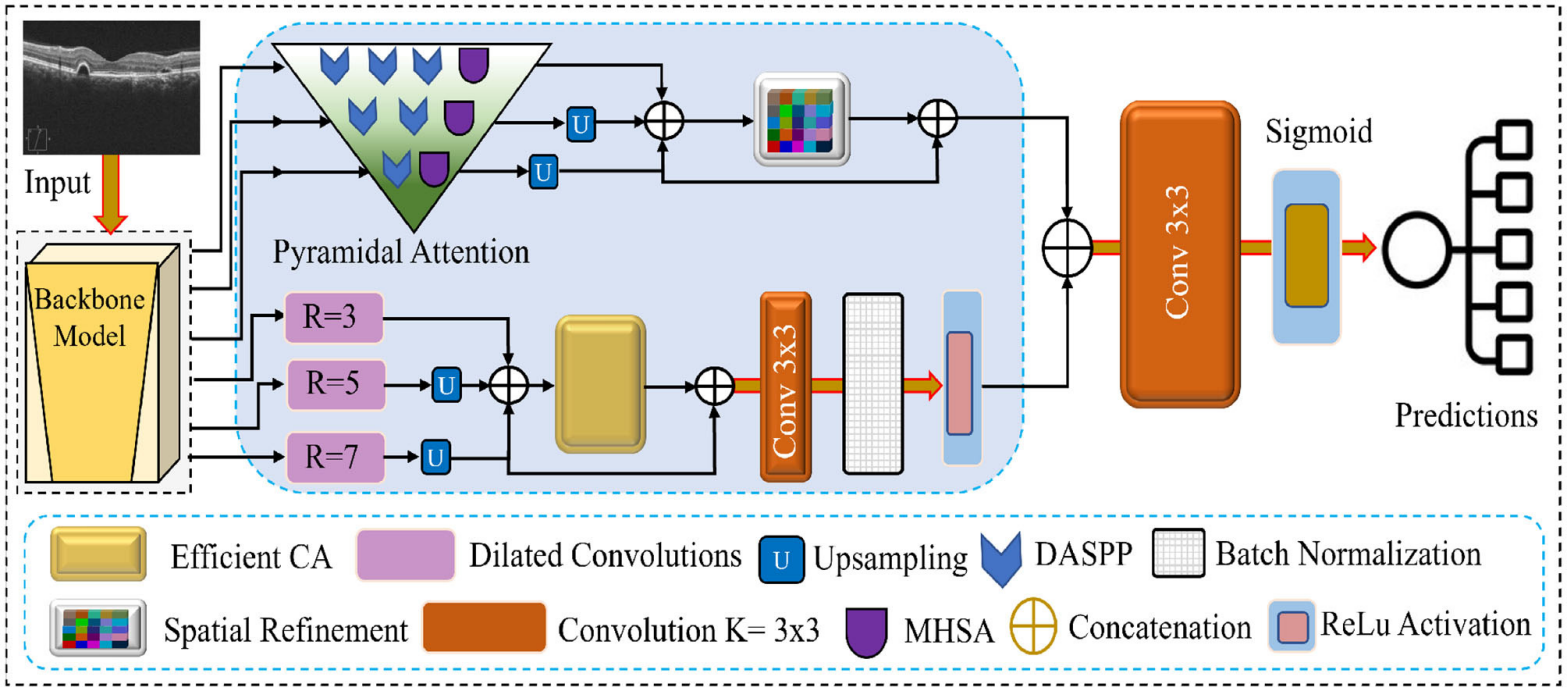
Visual overview of the features flow and the modules integrated into the proposed network.

This section introduces the proposed network for retinal disease recognition using OCT images. Our method distinguishes itself by addressing the challenges of effective feature extraction and representation through a comprehensive approach. The architecture incorporates Multiscale Feature Extraction, Multi-Headed Self Attention, Enhanced Spatial Attention, Dilated Convolution Block, and Efficient Channel Attention. These components are unique to our proposed network, as illustrated in Figure 1 work in synergy to provide a robust and efficient framework for recognizing retinal diseases. The following subsections delve into each component and its role in improving the overall performance of the proposed network.

### 3.1 Multiscale feature extraction

The proposed network employs EfficientNet-B7 as the backbone for multiscale feature extraction. The network allows for the extraction of features at six different scales, providing different scale features. Retinal structures and pathologies can manifest at various levels of detail, and this multiscale approach captures fine-grained textures and edges at lower levels, as well as complex structural patterns and global context at higher levels. This adaptability is essential for addressing the unique challenges of OCT images, including speckle noise, varying tissue reflectivity, and complex layer structures. By extracting a comprehensive set of features from multiple scales, our model ensures a thorough representation of the OCT image, which is essential for detecting subtle retinal abnormalities that might be visible only at specific scales or require context from multiple scales for accurate interpretation. The multiscale feature extraction from EfficientNet-B7 offers several key benefits for OCT image analysis. It balances accuracy and efficiency, which is crucial when processing large volumes of high-resolution images. The diverse feature set obtained enhances the sensitivity and specificity of the network, allowing for the detection of small, localized abnormalities and broader structural changes in the retina. This approach provides robustness against variabilities in OCT images due to factors like imaging equipment, patient movement, or ocular opacities. Furthermore, it facilitates fine-grained recognition, enabling the network to discern subtle differences crucial for the classification of retinal diseases. By establishing a strong foundation for advanced OCT image analysis, our multiscale feature extraction significantly enhances the ability of the network to capture complex patterns effectively.

Let *I* denote the input OCT image. The multiscale feature extraction process can be formulated as follows:


(1) $$\begin{array}{l} F_{i}=\phi_{i}\left(I\right),\;i\in 1,2,...,6 \end{array}$$

where *F*_*i*_ represents the feature map extracted at scale *i*, and ϕ_*i*_(·) denotes the function that maps the input image to the *i*-th scale feature space through the corresponding layers of EfficientNet-B7. The set of multiscale features F is then defined as:


(2) $$\begin{array}{l} F=F_{1},F_{2},...,F_{6} \end{array}$$

Each *F*_*i*_ captures different levels of abstraction:

- *F*_1_, *F*_2_: Low-level features (e.g., edges, textures)
- *F*_3_, *F*_4_: Mid-level features (e.g., layer boundaries, small structures)
- *F*_5_, *F*_6_: High-level features (e.g., global retinal structure, large pathologies)

This multiscale representation F forms the basis for subsequent processing in our proposed network, enabling comprehensive analysis of retinal structures and pathologies across various scales of abstraction in OCT images.

### 3.2 Pyramidal attention with MHSA and DASPP modules

The Pyramidal Attention mechanism in our proposed network refines features at multiple granularity levels to enhance local and global feature representation. This mechanism operates on three feature maps generated by the EfficientNetB7 backbone, refining them progressively through a combination of MHSA and DASPP modules. By doing so, the network captures multiscale contextual information and long-range dependencies, which are critical for detecting subtle retinal abnormalities in OCT images. The Pyramidal Attention mechanism processes the three feature maps as follows:

- **First feature map**: This is the least mature feature map, containing more fine-grained and local information. We apply three DASPP modules parallel to one MHSA module to enhance this map. The three DASPPs capture contextual information at varying dilation rates, allowing the network to gather multiscale features from different receptive fields. MHSA further refines the features by enabling the network to focus on relevant regions across the map, ensuring that local details and broader patterns are preserved.
- **Second feature map**: As this feature map is more mature than the first one, it is processed through two DASPP modules and the MHSA module. The two DASPP modules capture features at different scales, while the MHSA focuses on the most important regions. This combination ensures that the intermediate features are refined and contextual information from various scales is strengthened.
- **Third feature map**: The third feature map is the most mature and contains the highest level of abstraction. We passed these features from one DASPP and a single MHSA module to avoid over-processing these features. The single DASPP helps capture any remaining multiscale features, while the MHSA ensures the attention mechanism enhances critical areas of the feature map. This minimal refinement prevents the loss of global structural information while enhancing the most relevant feature regions.

#### 3.2.1 Dense Atrous Spatial Pyramid Pooling

DASPPs are the specialized variant of the Atrous Spatial Pyramid Pooling (ASPP) module, which is essential in our multiscale feature extraction (39). DASPP utilizes multiple 3 3 dilated convolutions with varying dilation rates to extract features from different receptive fields. This allows the network to gather contextual information from both fine and coarse details within the OCT images. In contrast to traditional ASPP, DASPP enhances the extracted features by fusing the input with the output through residual connections, which preserves important low-level features while introducing additional multiscale context.

#### 3.2.2 Multi-head self-attention

MHSA is applied at each level of feature refinement to capture long-range dependencies and to allow the network to focus on the most informative regions of the image. By attending to multiple areas in parallel, MHSA helps prioritize crucial retinal structures, such as lesions or tissue abnormalities, that may be subtly dispersed across the image. This attention mechanism is particularly useful for OCT images, where disease manifestations vary significantly in scale and location.

#### 3.2.3 Pyramidal refinement process

Combining DASPP and MHSA in a pyramidal structure ensures that feature refinement is conducted progressively. The DASPP and MHSA modules extract and integrate information from multiple scales at each stage. By applying different numbers of DASPP modules based on the maturity of the feature maps, our network efficiently processes both local details and global structures, resulting in a well-balanced representation critical for accurate disease classification. The overall refinement structure of the Pyramidal Attention block is illustrated in the following matrix:


$$\left[\begin{array}{cccc} \text{DASPP} & \text{DASPP} & \text{DASPP} & \text{MHSA}\\ \text{DASPP} & \text{DASPP} & \text{MHSA}\\ \text{DASPP} & \text{MHSA} \end{array}\right]$$

This structure ensures that each feature map is refined appropriately, enhancing the network's ability to detect small, localized features and larger, global patterns within the OCT images.

### 3.3 Enhanced spatial attention

After the pyramidal attention mechanism processes the multi-scale features from the backbone network, the resulting feature maps are upsampled to recover the spatial resolution of the original input. Following the upsampling, these features are passed to the spatial attention module for additional enhancement. The customized spatial attention module selectively emphasizes spatially significant regions in the feature maps. The second diagram shows that the input feature map *X*_α_, with dimensions *h*×*w*×*Ch*, is first subjected to max pooling operations to condense the most salient spatial information. The pooled features are then passed through two fully connected (FC) layers, interleaved with a non-linear activation function, to learn complex relationships between the spatial elements. At the end of this process, the sigmoid activation function generates the spatial attention map, which assigns weights to each spatial location based on its importance. These weights are subsequently used to modulate the input feature map *X*_α_, resulting in a weighted output $X_{ca}^{\prime }$. This refined feature map $X_{ca}^{\prime }$ carries spatially enhanced information, making it more discriminative for retinal disease classification in OCT images.

### 3.4 Dilated convolution block

In our proposed network, the fourth, fifth, and sixth feature maps extracted from the backbone network are processed using separate dilated convolution (DConv) blocks to capture context at multiple scales. Each of these dilated convolution blocks is designed with a 3 × 3 kernel size but operates with different dilation rates to expand the receptive field without losing spatial resolution. This multi-scale design enables the network to extract features that vary in granularity, which is crucial for effectively identifying fine and coarse retinal structures. The fourth feature map is passed through a DConv block with a dilation rate of 3. This rate allows the network to capture local features with a moderately expanded receptive field, preserving the details of smaller structures. The fifth feature map is processed through a DConv block with a dilation rate of 5. Finally, the sixth feature map is passed through a DConv block with a dilation rate of 7. This larger dilation rate enables the network to capture a broader context, which is essential for recognizing large-scale structures or more global patterns. The network effectively captures multi-scale features by applying different dilation rates to the fourth, fifth, and sixth feature maps. These features are then further refined and concatenated with the outputs of the pyramidal attention and spatial attention modules. This approach allows the network to combine fine-grained local details with larger contextual information, improving retinal disease recognition.

### 3.5 Efficient channel attention

**Figure 2:**
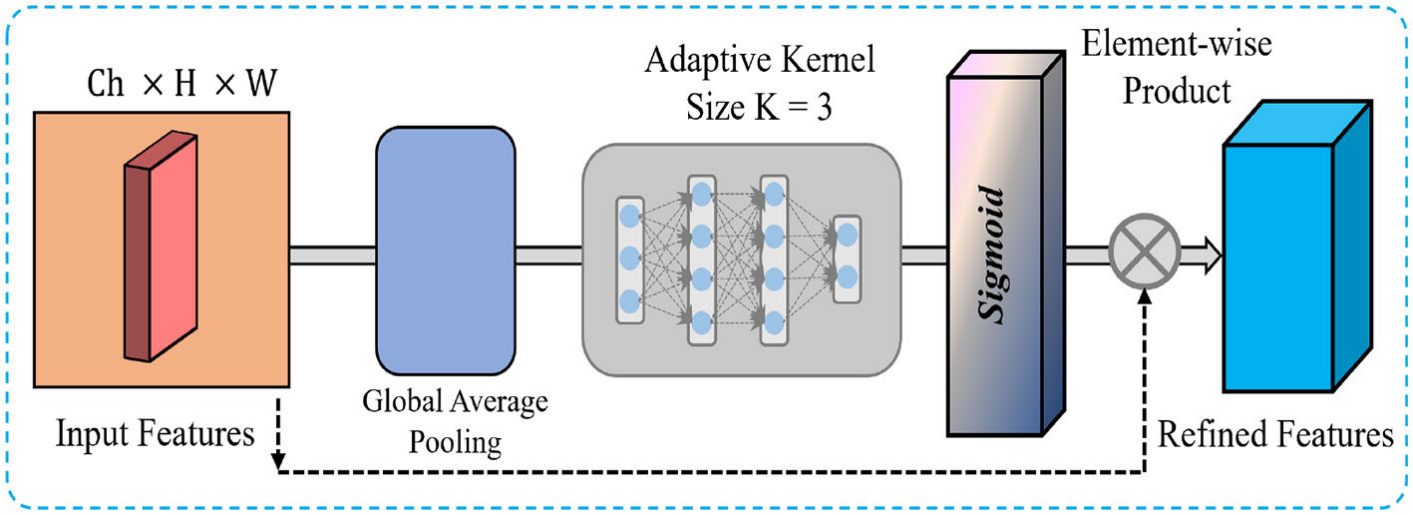
Visual overview of the features flow inside the efficient channel attention (ECA).

Subsequent to the processing of feature maps by the Dilated Convolution Blocks, these are upsampled and concatenated to generate a cohesive multi-scale feature representation. This concatenation effectively combines the information extracted at various dilation rates, capturing fine-grained details and larger-scale contextual information. We apply an ECA mechanism as shown in Figure 2 to enhance the most informative features while reducing computational overhead. The ECA module selectively emphasizes important channels by adaptively recalibrating the channel-wise dependencies, which helps the network focus on the most relevant features. In our network, we reduce the kernel size to 3, decreasing the computational complexity and ensuring that the attention mechanism remains efficient and lightweight. The ECA process begins by applying global average pooling to the concatenated feature maps, which generates a channel-wise descriptor. This descriptor is passed through fully connected layers and non-linear activation to produce channel attention weights. These weights are applied to the original concatenated feature maps via element-wise multiplication, effectively enhancing the important channels while suppressing less informative ones. By integrating the ECA module after the concatenated features, we ensure that the network efficiently focuses on the most important features, significantly improving both the computational efficiency and the feature representation for the subsequent layers. This enhancement is particularly critical in OCT image analysis, where identifying subtle yet crucial structures is essential for accurate retinal disease classification.

## 4 Experimental results

This section highlights the empirical setup, followed by the features of the utilized datasets, empirical validation of the framework, and the assorted factors. Subsequently, ablation experiments are conducted to thoroughly assess the effectiveness and efficiency of the modified and combined distinct attention systems. Finally, comparisons are conducted with prominent SOTA methodologies.

### 4.1 Experimental setup

Our experiments were conducted on a high-performance computing system to ensure efficient model training and evaluation. The hardware configuration consisted of an Intel (R) Core (TM) i9-10900X CPU with ten cores, complemented by 192 GB of system RAM. We employed an NVIDIA GeForce RTX 4090 GPU for accelerated DL computations, which offers 24 GB of VRAM. The software environment was based on Windows 10, with PyTorch serving as the primary DL framework. We utilized several essential Python libraries to support our research, including Scikit-learn for ML utilities, NumPy for numerical computations, Seaborn and Matplotlib for data visualization, tqdm for progress tracking, Pandas for data manipulation, and Pillow for image processing. Through extensive experimentation, we optimized various hyperparameters to achieve the best performance. The final configuration included 100 epochs and an input image resolution of 224 × 224 × 3. We chose a batch size of 8 to balance performance and memory constraints. We used the SGD optimizer with a momentum of 0.9, a learning rate of 0.0001, and a weight decay of 0.0005 for optimization. These parameters were selected based on empirical testing and insights from relevant literature in the field. The chosen setup provided an optimal trade-off between model performance and computational efficiency, allowing us to train and evaluate our models on the given datasets effectively.

### 4.2 Dataset

**Figure 3:**
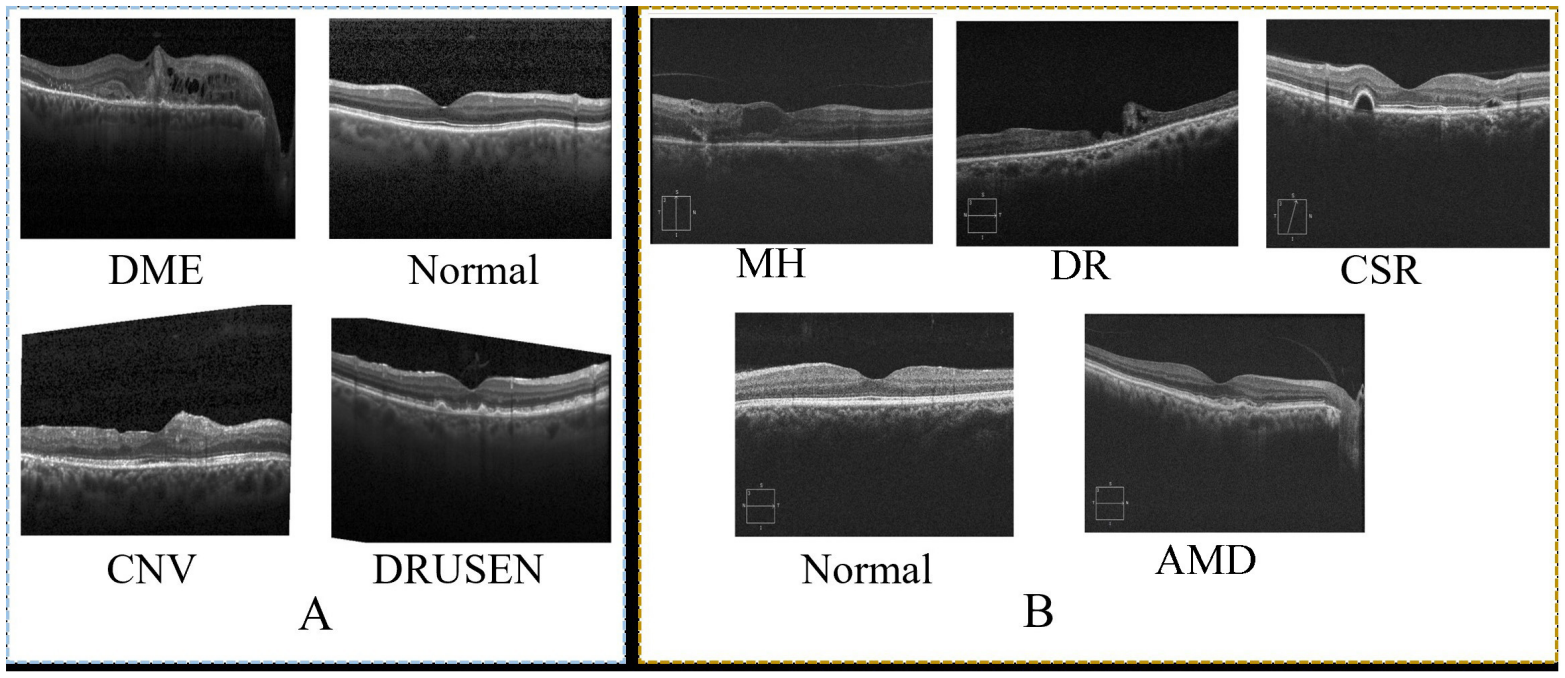
Sample of images from two datasets. **(A)**, shows sample from OCT (31) and **(B)** shows sample of images from OCTID (40).

This study used two prominent optical coherence tomography (OCT) datasets to train and evaluate our network. Table 2 provides an overview of these datasets, detailing the number of classes and train/test splits. Images from the datasets are shown in Figure 3. The dataset presented by Kermany et al. (31) is a comprehensive OCT image collection that has successfully established the collection as a standard benchmark in the domain. The dataset includes 109,312 training images grouped into four unique categories. This comprehensive collection is a solid basis for training models, with diverse OCT scans depicting various retinal diseases. Furthermore, the OCT image dataset (OCTID) (40) is a specialized dataset encompassing a wider array of retinal conditions. The dataset has 458 training visuals and 115 testing images categorized into five types. Although smaller than the Kermany dataset, OCTID provides variability in the represented circumstances, facilitating more complex classification tasks.

**Table 2:** Descriptions of the datasets include the number of classes, total images, and train/test splits adopted for our analysis.

**Dataset**	**Classes**	**Total images**	**Training images**	**Testing images**
OCT (31)	4	109,312	87,450 (80%)	21,862 (20%)
OCTID (40)	5	573	458	115

### 4.3 Evaluation metrices

We executed a broad range of tests to evaluate the classification performance of our suggested network thoroughly. The experiments included juxtaposing our network with several state-of-the-art methodologies using well-established metrics for evaluation. Our evaluation focused on three principal performance indicators: accuracy, sensitivity, and specificity. These indicators were selected to provide an extensive viewpoint of our network's ability. These measures facilitate a detailed understanding of the performance in numerous classification areas. Accuracy is a comprehensive metric for right predictions, while sensitivity and specificity elucidate the model's ability to identify positive and negative instances accurately. We computed these indicators using demonstrated mathematical formulas to ensure accuracy and comparability. These equations convert the raw classification results into measurable performance metrics, facilitating an objective comparison between our proposed model and the SOTA methodologies.


(3) $$\begin{array}{l} Accuracy=\frac{Num\;of\;correct}{Total} \end{array}$$

(4) $$\begin{array}{l} Precision=\frac{TP}{TP+FP} \end{array}$$

(5) $$\begin{array}{l} Recall/Sensitivity=\frac{TP}{TP+FN} \end{array}$$

### 4.4 Ablation study

Several network settings are examined for the comprehensive ablation study using the Kermany dataset to show module gradual and effective integration. We Started with the initial models and progressed to more sophisticated designs with attention mechanisms and feature refinement modules. The findings of the ablation investigation are shown in Table 3, and each module is discussed in the below subsections.

**Table 3:** Abalation study of the intermediate features with the integration of different modules and progressive features fusion.

**Network design**	**Accuracy (%)**	**Precision (%)**	**Recall (%)**	**F1 (%)**
**EffNet-B6**	93.20	92.73	93.30	92.15
**EffNet-B7**	94.02	93.88	94.98	94.42
**EffNet-B7 + PAttV1**	95.55	94.28	94.65	95.78
**EffNet-B7 + PAttV2**	95.98	95.62	94.92	94.88
**EffNet-B7 + PAttV2 + DConv**	96.18	95.81	96.18	96.02
**EffNet-B7 + PAttV2 + SA + DConv**	97.02	96.72	97.20	96.75
**EffNet-B7 + PAttV2 + SA + DConv + ECA**	98.10	97.91	98.02	98.20
**Proposed network**	98.74
	98.51
	97.34
	98.30

#### 4.4.1 Baseline networks (EffNet-B6 and EffNet-B7)

The analysis begins with EfficientNet-B6 as the backbone, achieving an accuracy of 93.20%, with precision, recall, and F1 scores in the 92-93% range. This strong baseline indicates that EfficientNet-B6 can perform well in extracting complex features. However, the results showed room for further improvement in capturing more complex features. When upgrading to EfficientNet-B7, the results significantly improve, with the accuracy increasing to 94.02%. This improvement is consistent across all metrics, reflecting that a more advanced backbone architecture provides better feature extraction capabilities, directly contributing to overall performance. However, the extracted features from EfficientNet-B7 need more refinement and attention using advanced attention mechanisms, which are crucial for better recognition performance.

#### 4.4.2 Effect of pyramid attention and dilated convolution

Introducing Pyramid Attention Version 1 (PAttV1), which incorporates multiscale attention using each DASPP and MHSA, significantly enhances the performance. The accuracy jumps to 95.55%, and the F1 score reaches 95.78%, demonstrating that pyramid attention effectively captures multiscale features, improving both precision and recall. PAttV1's ability to integrate information from various scales, particularly using different pyramid attention structures for the first three layers of EfficientNet-B7, allows the network to handle the inherent complexity of retinal patterns. Further improvement is seen with the introduction of Pyramid Attention Version 2 (PAttV2), which refines the attention mechanisms by increasing the number of DASPPs in the pyramid attention blocks (Final Pyramid block). The accuracy rises to 95.98%, and precision reaches 95.62%. This illustrates that PAttV2's deeper and more sophisticated attention structure allows the network to capture more nuanced spatial features, resulting in better detection outcomes. The addition of dilated convolution (DConv) blocks, which target deeper features from EfficientNet-B7's later layers (4, 5, and 6), further enhances performance. The accuracy improves to 96.18%, showcasing the importance of incorporating multiscale context through dilation rates of 3, 5, and 7. This setup allows the network to extract features across varying scales, leading to more comprehensive spatial awareness and improved detection.

#### 4.4.3 Impact of spatial attention and efficient channel attention

When SA is added alongside PAttV2 and DConv, the performances are substantially boosted, with accuracy increasing to 97.02% and the F1 score reaching 96.75%. Including spatial attention enhances the ability to focus on the most relevant regions of the feature maps, refining the spatial dependencies. By incorporating ECA on top of PAttV2, SA, and DConv, the accuracy reaches 98.10%. ECA reduces the computational cost by decreasing the kernel size, allowing for efficient refinement of channel-wise attention. This step is crucial as it balances computational efficiency with improved feature representation, enabling the network to capture essential information while reducing redundant features. Finally, the proposed network, which integrates all these components-EfficientNet-B7, PAttV2, SA, DConv, and ECA with convolution layer achieves the highest performance in the study, with an accuracy of 98.74%, precision of 98.51%, recall of 97.34%, and an F1 score of 98.30%. This demonstrates that the combined effect of multiscale attention, spatial and channel attention mechanisms, and dilated convolution blocks leads to a highly robust and accurate network.

### 4.5 Impact of the attention heads

**Figure 4:**
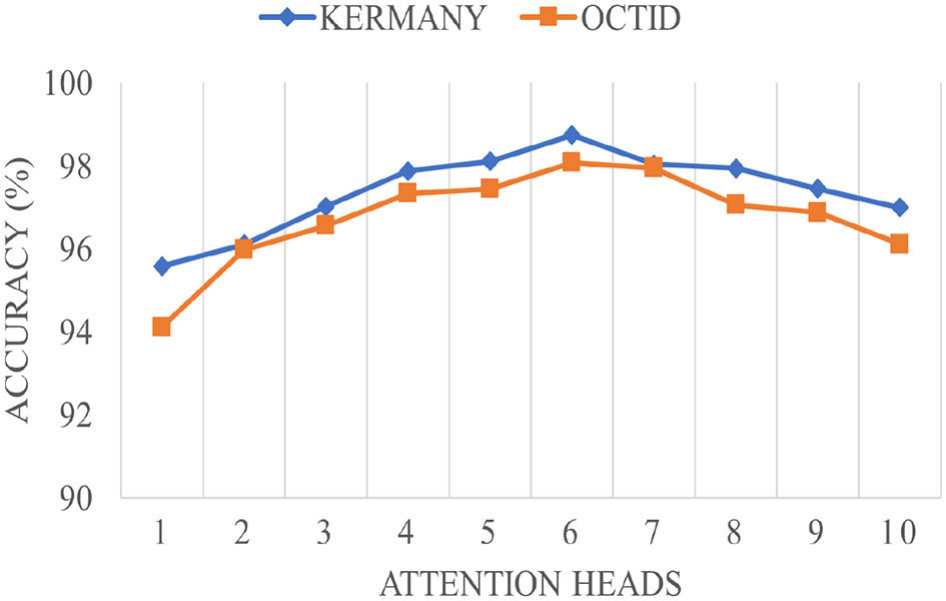
The impact of the attention heads in the MHSA module on the overall performance of the proposed network.

Figure 4 demonstrates the impact of varying the number of attention heads in the MHSA mechanism on the overall accuracy for Kermany and OCTID datasets. The accuracy trend follows a non-linear pattern as attention heads increase to 10. For both datasets, the accuracy increases steadily with the number of attention heads up to 6 heads, reaching peak performance around this point. The Kermany dataset attains a maximum accuracy of roughly 98.5%, whereas the OCTID dataset gets around 97.5% accuracy with six attention heads. This pattern indicates that growing the number of attention heads early enhances the capacity to discern more complex features, resulting in improved performance. However, after the top (6 attention heads), both datasets show a modest performance decrease. The Kermany dataset exhibits a decline in accuracy, reaching about 96% with ten attention heads, but the OCTID dataset similarly falls below 96% at the same confluence. This reduction can be attributed to probable overfitting and excessive complexity resulting from an abundance of attention heads, which led the network to neglect the most critical properties. The study indicates that six attention heads provide the ideal equilibrium between performance and complexity for both datasets.

### 4.6 Comparative analysis with SOTA methods

The comparative examination of numerous SOTA approaches in analysis demonstrates a consistent improvement in performance in recent years, as shown in Table 4. For instance, Rahimzadeh and Mohammadi (21) improved performance with a deep ensemble CNN reaching 96.4%, which was further matched by Subramanian et al. (22) using models like VGG16, VGG19, Densenet201, and InceptionV3, attaining 97% accuracy. More recent works, such as (4) deep CNN based on Inception architecture, achieved 95.55%, while Hassan et al. (6), and Diao et al. (23) obtained similar accuracies of 96.93% using random forest models with ResNet 50 and mask-guided CNNs, respectively. The Capsule network with Adaptive Histogram Equalization by Opoku et al. (5) and the Hybrid multilayered CNN-VGG19 model by Udayaraju et al. (7) both achieved 97.7% accuracy, showcasing the potential of hybrid architectures. Naik et al. (24) explored self-attention mechanisms with InceptionV3 and Xception, reaching 96.6%, though they still needed to surpass the top-performing models. In contrast, the proposed network outperformed all prior models with a remarkable accuracy of 98.74% on the Kermany dataset. This highlights the effectiveness of multi-scale feature extraction and attention mechanisms in capturing critical patterns within OCT images, leading to superior performance.

**Table 4:** Comparative analysis with the performances of the SOTA models on Kermany dataset.

**Method**	**References**	**Accuracy %**	**Year**
Deep ensemble CNN	(21)	96.4	2021
VGG16, VGG19, Densenet201, InceptionV3	(22)	97	2022
Deep CNN based on Inception architecture	(4)	95.55	2023
Random forest models with ResNet 50	(6)	97.47	2023
Mask guided CNN	(23)	96.93	2023
Capsule network with Adaptive histogram equalization	(5)	97.7	2023
Hybrid multilayered classification CNN-VGG19	(7)	97.7	2023
InceptionV3 and Xception with self-attention	(24)	96.6	2024
**Multi-scale features with attentions**	**The proposed (Ours)**	**98.74**	**2024**

## 5 Discussion

**Figure 5:**
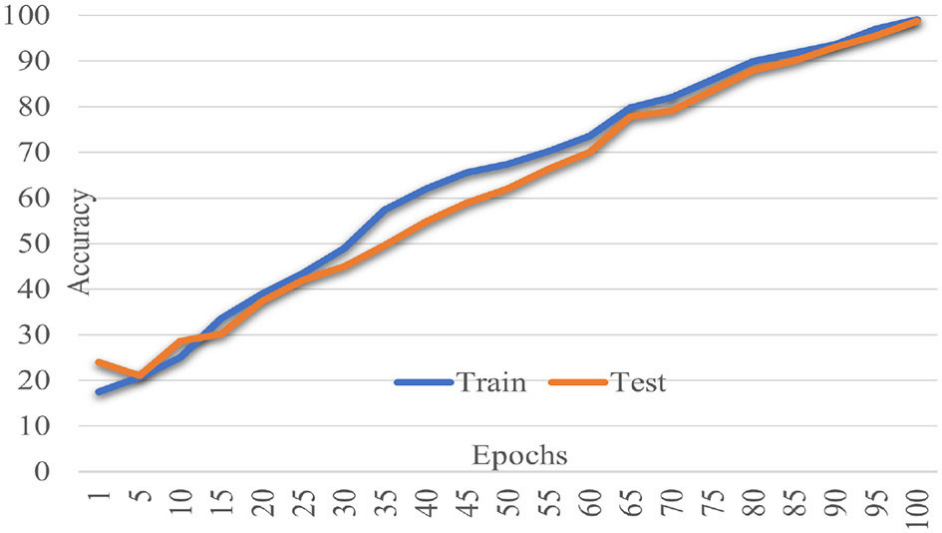
Model performance metrics on the Kermany dataset across 100 training epochs.

**Figure 6:**
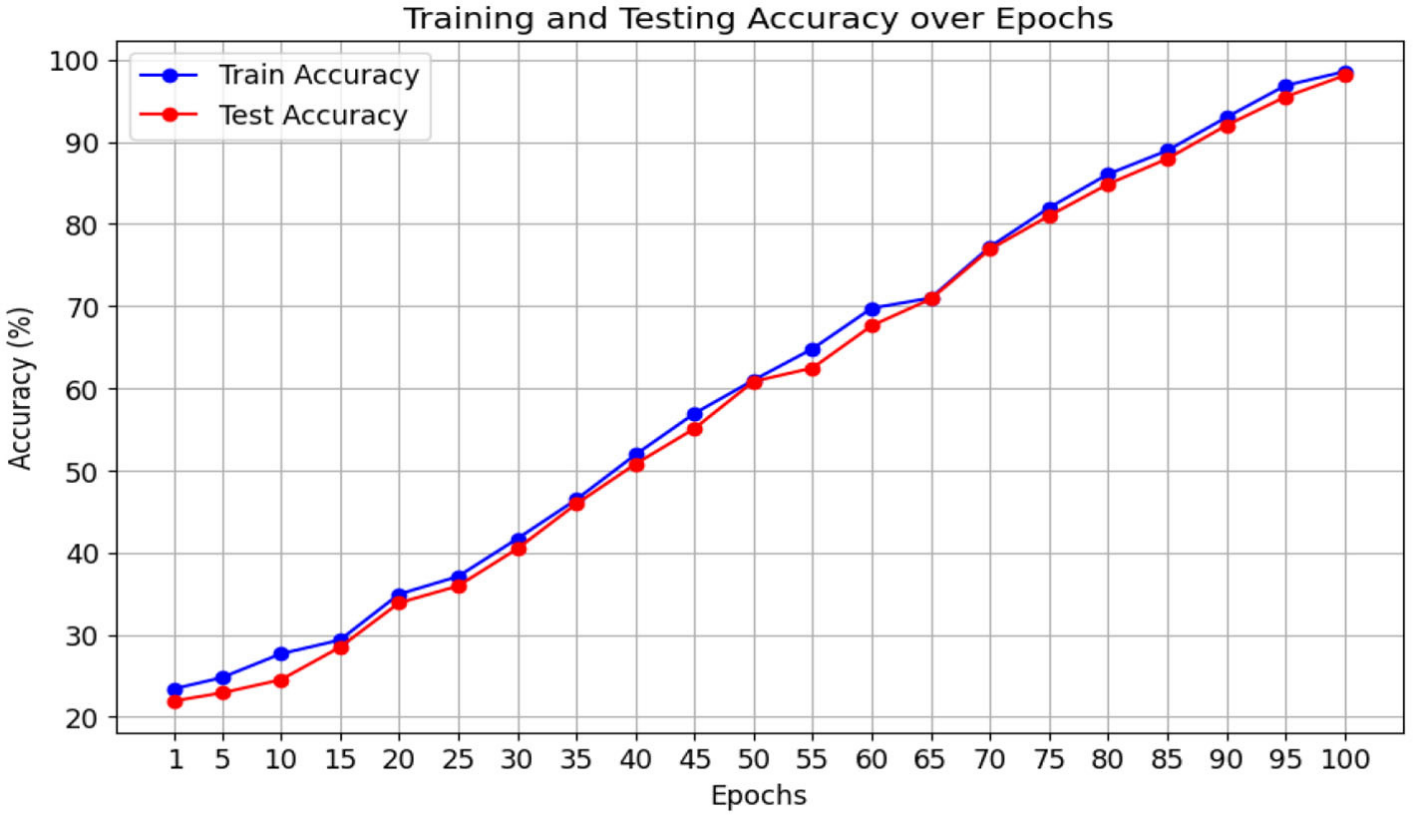
Model performance metrics on the OCTID dataset over 100 training epochs.

The proposed Multiscale Feature Enhancement via an Attention-over-Attention network addresses key challenges in retinal disease recognition from OCT images by leveraging advanced attention mechanisms and multiscale feature extraction techniques. Integrating the proposed network with an EfficientNetB7 backbone enables the extraction of comprehensive multiscale features that provide a detailed representation of fine-grained and global retinal structures, which are critical for accurate classification. Incorporating a Pyramidal attention mechanism, combining MHSA with DASPP, enhances the capacity of the network to capture long-range dependencies and contextual information at multiple scales, overcoming the limitations of existing methods that often rely on single-scale processing. This unique combination allows the network to focus on the most relevant features, ensuring precise localization of retinal abnormalities that traditional approaches might overlook. Furthermore, our design of parallel processing paths with varying dilation rates allows for concurrently analyzing multiple receptive fields, significantly improving the network's ability to distinguish between local details and global contextual cues, which is crucial for effective retinal disease recognition. The performance of our network was rigorously evaluated on two benchmark datasets: the Kermany dataset and the OCTID dataset, as shown in Figures 5, 6. In Figure 5, the performance metrics, including accuracy and loss, demonstrate consistent improvement over 100 training epochs, highlighting the ability to learn and generalize from the data effectively. Similarly, in Figure 6, the performance metrics on the OCTID dataset indicate robust learning dynamics, with steady convergence and high classification accuracy achieved across the training epochs. These results underscore the adaptability of our network to different datasets and its effectiveness in capturing complex retinal structures and abnormalities. The integration of ECA and Spatial Refinement modules further strengthens the network's performance by enhancing channel-wise and spatial feature representations, reflected in the superior metrics achieved across both datasets. Ablation studies confirm the critical roles of each component, validating our design choices and highlighting the advantages of advanced attention mechanisms and multiscale processing. These results demonstrate the potential of our approach to revolutionize OCT image analysis and provide ophthalmologists with more precise diagnostic tools for early detection and treatment planning.

## 6 Conclusion

In this study, we presented a novel deep learning Multiscale Feature Enhancement via Attention-over-Attention network for the recognition of retinal diseases using OCT images. Our approach addresses the existing limitations of continusely relyig on single scale features. The proposed network leveraged multiscale feature extraction, advanced attention mechanisms, and parallel processing paths to enhance the detection of subtle retinal abnormalities. The integration of the EfficientNetB7 backbone enables the network to extract multi-level features. These features are then refined by the feasible integration of attention mechanisms, which include the Pyramidal Attention mechanism, dilated convolution block, Efficient Channel Attention (ECA), and Spatial Refinement modules. These modules collectively contribute to the superior performance of the proposed network over extensive experiments. The proposed network achieved state-of-the-art accuracy and enhanced the interpretability and reliability of OCT image analysis, making it a promising tool. Future research may focus on further refining the attention mechanisms, exploring additional datasets for complex features, and adapting the model for real-time clinical deployment to support ophthalmologists in early and precise diagnosis of retinal diseases.

## Data Availability

Publicly available datasets were analyzed in this study. This data can be found here: Kermany (31) and OCTID (40) public datasets.

## References

[1] World Health Organization. Blindness and vision impairment (2023). Available at: https://www.who.int/news-room/fact-sheets/detail/blindness-and-visual-impairment

[2] Khan H Ullah I Shabaz M Omer MF Usman MT Guellil MS. Visionary vigilance: optimized YOLOV8 for fallen person detection with large-scale benchmark dataset. Image Vis Comput. (2024) 149:105195. 10.1016/j.imavis.2024.105195

[3] Díaz M Novo J Cutrín P Gómez-Ulla F Penedo MG Ortega M. Automatic segmentation of the foveal avascular zone in ophthalmological OCT-A images. PLoS ONE. (2019) 14:e0212364. 10.1371/journal.pone.0212364 30794594 PMC6386246

[4] Stanojević M Drašković D Nikolić B. Retinal disease classification based on optical coherence tomography images using convolutional neural networks. J Electr Imag. (2023) 32:032004. 10.1117/1.JEI.32.3.032004

[5] Opoku M Weyori BA Adekoya AF Adu K. CLAHE-CapsNet: efficient retina optical coherence tomography classification using capsule networks with contrast limited adaptive histogram equalization. PLoS ONE. (2023) 18:e0288663. 10.1371/journal.pone.0288663 38032915 PMC10688733

[6] Hassan E Elmougy S Ibraheem MR Hossain MS AlMutib K Ghoneim A. Enhanced deep learning model for classification of retinal optical coherence tomography images. Sensors. (2023) 23:5393. 10.3390/s23125393 37420558 PMC10301292

[7] Udayaraju P Jeyanthi P Sekhar B. A hybrid multilayered classification model with VGG-19 net for retinal diseases using optical coherence tomography images. Soft Comput. (2023) 27:12559–70. 10.1007/s00500-023-08928-w

[8] Khalil I Mehmood A Kim H Kim J. OCTNet: a modified multi-scale attention feature fusion network with inception V3 for retinal OCT image classification. Mathematics. (2024) 12:3003. 10.3390/math12193003

[9] Khan H Jan Z Ullah I Alwabli A Alharbi F Habib S. A deep dive into AI integration and advanced nanobiosensor technologies for enhanced bacterial infection monitoring. Nanotechnol Rev. (2024) 13:20240056. 10.1515/ntrev-2024-0056

[10] Khan H Ullah M Al-Machot F Cheikh FA Sajjad M. Deep learning based speech emotion recognition for Parkinson patient. Electr Imag. (2023) 35:298–1. 10.2352/EI.2023.35.9.IPAS-29827534393

[11] Ur Rehman I Ullah I Khan H Guellil MS Koo J Min J. A comprehensive systematic literature review of ML in nanotechnology for sustainable development. Nanotechnol Rev. (2024) 13:20240069. 10.1515/ntrev-2024-0069

[12] Tufail AB Ullah I Khan WU Asif M Ahmad I Ma YK. Diagnosis of diabetic retinopathy through retinal fundus images and 3D convolutional neural networks with limited number of samples. Wirel Commun Mobile Comput. (2021) 2021:6013448. 10.1155/2021/6013448

[13] Oliveira J Gonçalves L Ferreira M Silva CA. Drusen detection in OCT images with AMD using random forests. In: 2017 *IEEE 5th Portuguese Meeting on Bioengineering (ENBENG)*. IEEE (2017). p. 1–4. 10.1109/ENBENG.2017.7889444

[14] Habib S Alyahya S Ahmed A Islam M Khan S Khan I. X-ray image-based COVID-19 patient detection using machine learning-based techniques. In: Computer Systems Science & Engineering. Tech Science Press (2022). p. 671–682. 10.32604/csse.2022.021812

[15] Asgari R Orlando JI Waldstein S Schlanitz F Baratsits M Schmidt-Erfurth U. Multiclass segmentation as multitask learning for drusen segmentation in retinal optical coherence tomography. In: Medical Image Computing and Computer Assisted Intervention-MICCAI 2019: 22nd International Conference, Shenzhen, China, October 13-17, 2019, Proceedings, Part I 22. Springer (2019). p. 192–200. 10.1007/978-3-030-32239-7_22

[16] Wang M Zhu W Shi F Su J Chen H Yu K. MsTGANet: automatic drusen segmentation from retinal OCT images. IEEE Trans Med Imaging. (2021) 41:394–406. 10.1109/TMI.2021.3112716 34520349

[17] Rasti R Rabbani H Mehridehnavi A Hajizadeh F. Macular OCT classification using a multi-scale convolutional neural network ensemble. IEEE Trans Med Imaging. (2017) 37:1024–34. 10.1109/TMI.2017.2780115 29610079

[18] Lu W Tong Y Yu Y Xing Y Chen C Shen Y. Deep learning-based automated classification of multi-categorical abnormalities from optical coherence tomography images. Transl Vision Sci Technol. (2018) 7:41–41. 10.1167/tvst.7.6.41 30619661 PMC6314222

[19] Aloraini M Khan A Aladhadh S Habib S Alsharekh MF Islam M. Combining the transformer and convolution for effective brain tumor classification using MRI images. Appl Sci. (2023) 13:3680. 10.3390/app13063680

[20] Kim J Tran L. Ensemble learning based on convolutional neural networks for the classification of retinal diseases from optical coherence tomography images. In: 2020 *IEEE 33rd International Symposium on Computer-Based Medical Systems (CBMS)*. IEEE (2020). p. 532–537. 10.1109/CBMS49503.2020.00106

[21] Rahimzadeh M Mohammadi MR. ROCT-Net: A new ensemble deep convolutional model with improved spatial resolution learning for detecting common diseases from retinal OCT images. In: 2021 11*th International Conference on Computer Engineering and Knowledge (ICCKE)*. IEEE (2021). p. 85–91. 10.1109/ICCKE54056.2021.9721471

[22] Subramanian M Shanmugavadivel K Naren OS Premkumar K Rankish K. Classification of retinal OCT images using deep learning. In: 2022 International Conference on Computer Communication and Informatics (ICCCI). IEEE (2022). p. 1–7. 10.1109/ICCCI54379.2022.9740985

[23] Diao S Su J Yang C Zhu W Xiang D Chen X. Classification and segmentation of OCT images for age-related macular degeneration based on dual guidance networks. Biomed Signal Process Control. (2023) 84:104810. 10.1016/j.bspc.2023.104810

[24] Naik G Narvekar N Agarwal D Nandanwar N Pande H. Eye disease prediction using ensemble learning and attention on OCT scans. In: Future of Information and Communication Conference. Springer (2024). p. 21–36. 10.1007/978-3-031-53960-2_3

[25] Yang J Wang G Xiao X Bao M Tian G. Explainable ensemble learning method for OCT detection with transfer learning. PLoS ONE. (2024) 19:e0296175. 10.1371/journal.pone.0296175 38517913 PMC10959366

[26] Xi X Meng X Qin Z Nie X Yin Y Chen X. IA-net: informative attention convolutional neural network for choroidal neovascularization segmentation in OCT images. Biomed Opt Expr. (2020) 11:6122–36. 10.1364/BOE.400816 33282479 PMC7687935

[27] Yasir M Ullah I Choi C. Depthwise channel attention network (DWCAN): an efficient and lightweight model for single image super-resolution and metaverse gaming. Expert Syst. (2024) 41:e13516. 10.1111/exsy.13516

[28] Zhang Y Ji Z Wang Y Niu S Fan W Yuan S. MPB-CNN: a multi-scale parallel branch CNN for choroidal neovascularization segmentation in SD-OCT images. OSA Continuum. (2019) 2:1011–27. 10.1364/OSAC.2.001011

[29] Meng Q Wang L Wang T Wang M Zhu W Shi F. MF-Net: Multi-scale information fusion network for CNV segmentation in retinal OCT images. Front Neurosci. (2021) 15:743769. 10.3389/fnins.2021.743769 34690681 PMC8533052

[30] Wang W Xu Z Yu W Zhao J Yang J He F. Two-stream CNN with loose pair training for multi-modal AMD categorization. In: Medical Image Computing and Computer Assisted Intervention-MICCAI 2019: 22nd International Conference, Shenzhen, China, October 13-17, 2019, Proceedings, Part I 22. Springer (2019). p. 156–164. 10.1007/978-3-030-32239-7_18

[31] Kermany DS Goldbaum M Cai W Valentim CC Liang H Baxter SL. Identifying medical diagnoses and treatable diseases by image-based deep learning. Cell. (2018) 172:1122–31. 10.1016/j.cell.2018.02.010 29474911

[32] Li C Wang M Sun X Zhu M Gao H Cao X. A novel dimensionality reduction algorithm for Cholangiocarcinoma hyperspectral images. Optics Laser Technol. (2023) 167:109689. 10.1016/j.optlastec.2023.10968937506600

[33] Arshad T Zhang J Ullah I. A hybrid convolution transformer for hyperspectral image classification. Eur J Remote Sensing. (2024) 2024:2330979. 10.1080/22797254.2024.233097939325597

[34] Fang L Wang C Li S Rabbani H Chen X Liu Z. Attention to lesion: Lesion-aware convolutional neural network for retinal optical coherence tomography image classification. IEEE Trans Med Imaging. (2019) 38:1959–70. 10.1109/TMI.2019.2898414 30763240

[35] Farman H Khan T Khan Z Habib S Islam M Ammar A. Real-time face mask detection to ensure COVID-19 precautionary measures in the developing countries. Appl Sci. (2022) 12:3879. 10.3390/app12083879

[36] Sotoudeh-Paima S Jodeiri A Hajizadeh F Soltanian-Zadeh H. Multi-scale convolutional neural network for automated AMD classification using retinal OCT images. Comput Biol Med. (2022) 144:105368. 10.1016/j.compbiomed.2022.105368 35259614

[37] Ma Z Xie Q Xie P Fan F Gao X Zhu J. HCTNet: a hybrid ConvNet-transformer network for retinal optical coherence tomography image classification. Biosensors. (2022) 12:542. 10.3390/bios12070542 35884345 PMC9313149

[38] Rahil M Anoop B Girish G Kothari AR Koolagudi SG Rajan J. A deep ensemble learning-based CNN architecture for multiclass retinal fluid segmentation in oct images. IEEE Access. (2023) 11:17241–51. 10.1109/ACCESS.2023.3244922

[39] Khan H Hussain T Khan SU Khan ZA Baik SW. Deep multi-scale pyramidal features network for supervised video summarization. Expert Syst Appl. (2024) 237:121288. 10.1016/j.eswa.2023.121288

[40] Gholami P Roy P Parthasarathy MK Lakshminarayanan V. OCTID optical coherence tomography image database. Comput Electr Eng. (2020) 81:106532. 10.1016/j.compeleceng.2019.106532

